# Dose distribution prediction for head-and-neck cancer radiotherapy using a generative adversarial network: influence of input data

**DOI:** 10.3389/fonc.2023.1251132

**Published:** 2023-09-26

**Authors:** Xiaojin Gu, Victor I. J. Strijbis, Ben J. Slotman, Max R. Dahele, Wilko F. A. R. Verbakel

**Affiliations:** ^1^ Department of Radiation Oncology, Amsterdam UMC Location Vrije Universiteit Amsterdam, Amsterdam, Netherlands; ^2^ Cancer Center Amsterdam, Cancer Treatment and Quality of Life, Amsterdam, Netherlands

**Keywords:** artificial intelligence, machine learning, deep learning, generative adversarial network (GAN), dose prediction, OAR sparing, radiotherapy

## Abstract

**Purpose:**

A three-dimensional deep generative adversarial network (GAN) was used to predict dose distributions for locally advanced head and neck cancer radiotherapy. Given the labor- and time-intensive nature of manual planning target volume (PTV) and organ-at-risk (OAR) segmentation, we investigated whether dose distributions could be predicted without the need for fully segmented datasets.

**Materials and methods:**

GANs were trained/validated/tested using 320/30/35 previously segmented CT datasets and treatment plans. The following input combinations were used to train and test the models: CT-scan only (C); CT+PTVboost/elective (CP); CT+PTVs+OARs+body structure (CPOB); PTVs+OARs+body structure (POB); PTVs+body structure (PB). Mean absolute errors (MAEs) for the predicted dose distribution and mean doses to individual OARs (individual salivary glands, individual swallowing structures) were analyzed.

**Results:**

For the five models listed, MAEs were 7.3 Gy, 3.5 Gy, 3.4 Gy, 3.4 Gy, and 3.5 Gy, respectively, without significant differences among CP-CPOB, CP-POB, CP-PB, among CPOB-POB. Dose volume histograms showed that all four models that included PTV contours predicted dose distributions that had a high level of agreement with clinical treatment plans. The best model CPOB and the worst model PB (except model C) predicted mean dose to within ±3 Gy of the clinical dose, for 82.6%/88.6%/82.9% and 71.4%/67.1%/72.2% of all OARs, parotid glands (PG), and submandibular glands (SMG), respectively. The R^2^ values (0.17/0.96/0.97/0.95/0.95) of OAR mean doses for each model also indicated that except for model C, the predictions correlated highly with the clinical dose distributions. Interestingly model C could reasonably predict the dose in eight patients, but on average, it performed inadequately.

**Conclusion:**

We demonstrated the influence of the CT scan, and PTV and OAR contours on dose prediction. Model CP was not statistically different from model CPOB and represents the minimum data statistically required to adequately predict the clinical dose distribution in a group of patients.

## Introduction

Head and neck cancer (HNC) radiotherapy treatment planning is complex, due to large and irregular planning target volumes (PTV), multiple prescription/PTV dose levels (e.g., primary tumor and nodal areas), and a large range of organs at risk (OARs) in close proximity to the PTVs. It requires extensive contouring of all relevant target and OAR structures on a planning computed tomography (CT) scan, which is a labor- and time-intensive process subject to inter- and intra-observer variation. The treatment planning process can take several hours to complete (1), and the dose distribution is dependent on the skills and experience of the planner and the institution (2, 3). In order to increase efficiency and reduce variation in quality, automated treatment planning technologies have been introduced in recent years (4–6).

Traditionally, automated approaches relied on modeling spatial relationships between target volumes and OARs (e.g., overlap volume histograms (7, 8), distance-to-target histograms) in combination with machine learning algorithms to identify correlations between predictive volumetric or spatial features and dosimetry. Important limitations of such knowledge-based planning approaches are the limited predictability in cases where the clinical situation is not adequately represented by the library of patient plans.

More recently, deep learning (DL) has been investigated for automated treatment planning by using convolutional neural networks (CNNs) that incorporate contextual information with precise localization to solve a wide variety of imaging-related problems (e.g., U-Net (9), ResNet (10)). Given the non-linearity of source inputs (e.g., CT, PTV, and OAR contours) and the target output (dose distribution), dose prediction may be regarded as an image synthesis task (11). While U-Net and its derivatives have been widely used for dose distribution prediction (12–17), generative adversarial networks (GANs) are a method to implicitly learn density functions that estimate the probability distribution from training data through adversarial learning. A GAN uses two concurrent generative and discriminative neural networks to generate realistic predictions (18, 19). The objective of the generative network is to increase the error rate of the discriminative network, whereas the discriminator tries to classify realism. As a result, the GAN learns features of a realistic dose distribution for given anatomical characteristics and may be more capable than CNN-based neural networks of predicting dose distributions (20–22). Therefore, for this piece of work, we have selected a GAN-based approach.

In most previous works (11, 20, 21, 23, 24), dose prediction was based on an input of CT, PTV contours, and OAR contours. In contrast, we investigated if clinically acceptable, realistic HNC dose distributions could be predicted from the patient CT and primary tumor and lymph node PTVs, without explicit prior knowledge of the relevant OARs, in comparison with CT scan with OAR contours. This could circumvent the laborious and error-prone process of OAR contouring and be of relevance to routine clinical care, and in other scenarios like the rapid selection of patients most suitable for proton therapy (25). In addition, we investigated the added value of the CT itself, and if the neural network (NN) could recognize the tumor and OARs in the CT without providing any contours. In total, five different models trained with different combinations of input data were evaluated.

## Materials and methods

### Data acquisition

The dataset consisted of 350 patients who had previously been treated for locally advanced HNC between 2013 and 2018, and 35 patients treated in 2019 that were used as an independent test set. Each patient had a treatment plan consisting of two full volumetric modulated arc therapy (VMAT) arcs, delivering 35 fractions of 2 Gy to PTV-boost (PTV-B) and 1.55 Gy to PTV-elective (PTV-E). All tumor sites were included. During the selected time period, plans were made with different versions of the Eclipse treatment planning system; however, they all consistently aimed to achieve a low mean dose to the individual salivary glands and swallowing structures. The volume receiving 95% of the prescribed dose (V95) was ≥99% for PTV-B and ≥98% for PTV-E. From 2013 to 2014, plans were made by manually interactively adapting OAR optimization objectives during optimization. From 2014 to 2017, plans were made using in-house-developed automated interactive optimization (AIO) software, which automatically performed what planners previously had to do manually (6, 26). From 2017, plans were made using RapidPlan (Varian, a Siemens Healthineers Company, Palo Alto, CA, USA), which used a model based on previous AIO-generated plans. It was previously shown that treatment plans improved over time (27). Oral cavity mean dose reduction was introduced around 2016 and intensified in 2019.

Each patient in the dataset contains a three-dimensional (3D) planning CT scan, structure set, and dose distribution. Patients had to have at least one OAR structure available out of all the individual salivary glands and swallowing structures (**Table 1**). CT acquisition resolutions were [0.80,1.27] mm in-plane and 2.5 mm longitudinal, and acquisition dimensions were 512 × 512 × [97,228] voxels. The dose distribution resolutions were 2.5 mm isotropic. OAR contours of salivary glands and swallowing structures were grouped and unified into composite salivary glands (CSG) and composite swallowing structures (CSS), respectively (**Table 1**). In total, six structures were used for model training, validation, and testing: CSG, CSS, spinal canal (SC), PTV-E, PTV-B, and body contour.

**Table 1:** Overview of the relevant structures and their percentage prevalence in the RT data set.

Structure	Occurrence
Composite salivary glands*	
Parotid gland (L/R)	99%
Submandibular gland (L/R)	83%
Composite swallowing structures*	
Lower larynx	76%
Upper larynx	68%
Cricopharynx	70%
Esophagus	61%
Trachea	60%
Thyroid	62%
Upper esophageal sphincter	73%
Inferior pharyngeal constrictor muscle	76%
Medial pharyngeal constrictor muscle	69%
Superior pharyngeal constrictor muscle	73%
Nervous structures	
Spinal canal*	98%
Brain stem	44%
Planning target volumes	
PTV-E*	100%
PTV-B*	100%
Individual structures	
Oral cavity	90%
Body*	100%

* used as single-channel 3D images for model training, validation, and testing. Composite salivary glands (CSG) and composite swallowing structures (CSS) were unified structures of the respective subsequent individual structures.

### Preprocessing

CT Hounsfield units were window-leveled from −200 to +300, similar to what would be used for head and neck automated segmentation tasks (28). Dose was capped at a maximum of 79 Gy. CTs and doses were normalized to [0,1], and structures were binarized as masks. To accommodate hardware limitations, the dataset was cropped from the original images. Based on the smallest number of slices for all patients, the most central 96 slices from each CT scan were selected and the data was cropped in left–right to retain the middle 256 out of 512 voxels (entire head and neck remained included, shoulders were removed). Then, in the vertical direction, 256 voxels starting from the tip of the nose were retained. Finally, this volume was resized to a 128 × 128 × 64 grid using trilinear and nearest-neighbor interpolation for the real-valued volumes (CT and dose) and binary valued structure masks, respectively, where the final voxel size was [1.60, 2.55] × [1.60, 2.55] × 3.75 mm. Cropping occasionally resulted in the loss of some caudal PTV and OAR containing slices resulting in the loss of 5% of OAR voxels, on average.

### Model architecture

**Figure 1:**
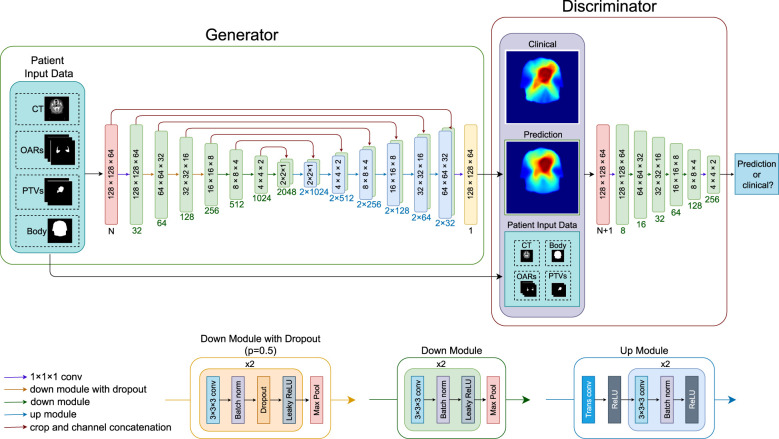
Overview of the neural network architecture for a forward pass. Blocks inside the generator and discriminator indicate the image dimensions (block sizes not in scale), numbers below the blocks indicate channel numbers. The neural network can take any combination of the patient input data, CT (one channel), OARs (three channels), PTV-E and PTV-B (two channels), and body contour (one channel), where N is the number of channels of the combined input data. LeakyReLU negative slope = 0.2, dropout rate = 0.5. CT, computed tomography; OAR, organ at risk; PTV, planning target volume; conv, convolutional layer; trans conv, transposed convolution; batch norm, batch normalization; ReLU, rectified linear activation unit.

**Figure 1** shows the architectures of the GAN. The generator is an adapted deep 3D U-Net. It can take any combinations of CT, PTVs (PTV-B and PTV-E), OAR structures, and the body contour as input and outputs a predicted dose distribution. The discriminator takes the same input channels as the generator together with the clinical dose distribution as training target and outputs the probability that the predicted dose cannot be distinguished from a clinical dose distribution. The discriminator is discarded after training; only the generator is used for dose prediction.

### Model training

The 350 patients in the dataset were randomly split into sets of 320 and 30 for training and validation, respectively. Model training was done on four NVIDIA GeForce GTX 2080 Ti graphics processor units (GPUs), each having 11 GB of GPU RAM, using PyTorch 1.11 and Python 3.9.16. The generator was trained using the ADAM optimizer (29) with β_1_ = 0.5 and β_2_ = 0.999, and the discriminator was trained with stochastic gradient descent (SGD). The initial learning rates were 0.001 for both networks, and a learning rate scheduler to decay by 10% every 20 epochs was applied to the generator. Batch size = 4 was the maximum number that could fit in the combined GPU memory. The conditional GAN’s objective is given by a weighted combination of the adversarial and reconstruction losses:


$$L_{cGAN}\left(G,D\right)=L_{adv}\left(G,D\right)+\lambda \;L_{rec}\left(G\right),$$

where the adversarial loss $L_{adv}\;$ is given by the binary cross-entropy loss (19) and the reconstruction loss $L_{rec}$ is a weighted combination of the 1 × L1 loss (mean absolute error) and 0.5 × L2 loss (mean squared error) functions, which we named elastic loss, motivated by the elastic net regularization. The weighting hyperparameter λ was chosen as 10, which gave the best empirical results among the values of 1, 10, and 100 we experimented with. For data augmentation, we used random horizontal flipping to increase the number of training samples.

After model development and hyperparameter tuning based on the evaluation of the validation set, five experiments were conducted. All experiments used the same NN architecture with the same hyperparameters, trained and tested on the same patients; the only difference was the patient information data used as input. The experiments were as follows: 1. Model C used only CT as input data, 2. Model CP used CT and PTVs. 3. Model CPOB used CT, PTVs, OARs, and body contour. 4. Model POB used PTVs, OARS, and body contour (but no CT). 5. Model PB used PTVs and body contour as input data (and no CT). All models were trained for 400 epochs with the same random seed. The composite OAR contours were used for model training to provide extra geometric information and were not used in the loss function.

Models were designed to answer the following questions: (C) How much can an NN learn when only the CT scan is provided as input? (CP-CPOB) Does the presence of the OAR contours in the training result in a statistically significant influence on dose prediction? (CPOB-POB) Does CT data offer significant improvements for the models? (PB) In case CT data makes a better model, does it learn from CT pixels where the OARs are, or does it estimate an approximate position of OARs based on all the average position of all training data?

### Evaluation

Predicted dose distributions for the 35 test patients by the five models trained with different input data were compared with doses from the clinical plans. The mean OAR dose and the mean absolute dose difference (MAE) in a volume of interest (VOI) were compared, 2D dose distributions were selected, and dose volume histograms (DVH) are presented. The VOI consisted of the part of the body for slices containing PTV-E, and where the PTV-E did not reach the cranial or caudal ends of the crop, two additional slices were added (7.5 mm). The VOI did not contain any background air and is the volume, which contains most of the dose. We also performed a significance test of the MAE in the VOI, to evaluate the statistical influence of the different types of input data. Wilcoxon significance test was used for each model tested against the other four models, α = 0.05, with a Bonferroni correction per set of tests to adjust the *p*-values.

**Figure 2:**
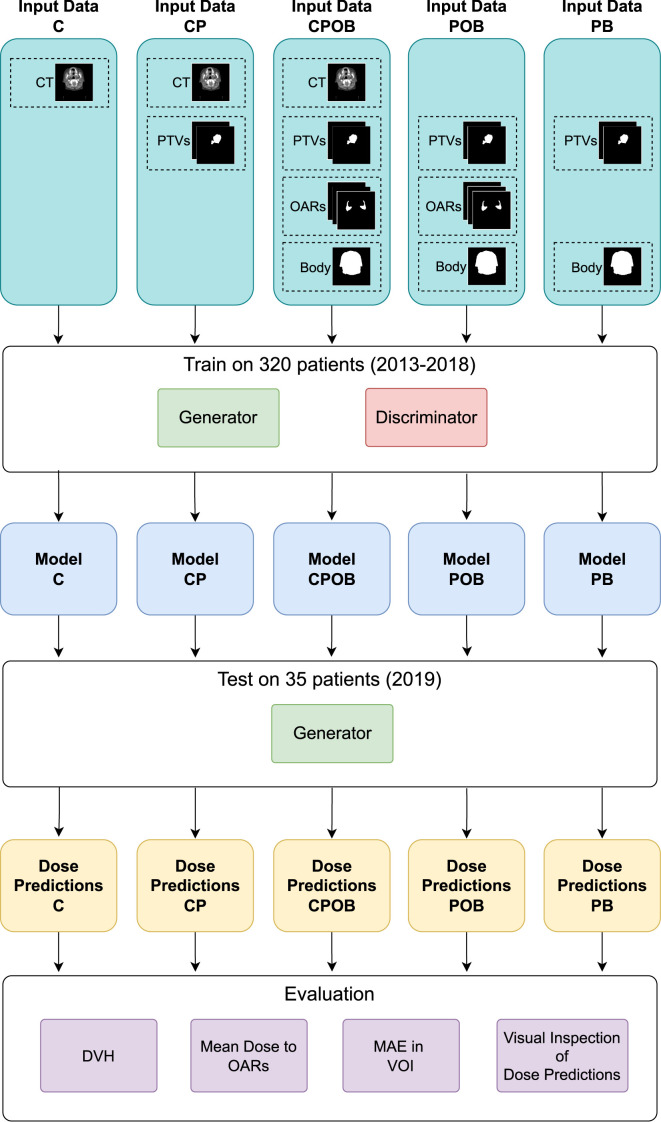
The flowchart of the experiments. The five combinations of the input data of CT, PTVs, OARs, and body contour were used to train the five models. Thereafter, the models were tested on the same test set to make the dose predictions. Finally, the predicted doses were evaluated by the metrics of dose volume histogram (DVH), mean dose to OARs, mean absolute error (MAE) in the volume of interest (VOI), and qualitative visual inspection on the predicted doses.

**Figure 2** shows the flowchart of the experiment setup. The different combinations of the input data were used to train different models. The five models were then tested on the same test set to make dose predictions. Finally, the five sets of prediction were evaluated based on the metrics.

## Results

### Dose volume histograms

**Figure 3:**
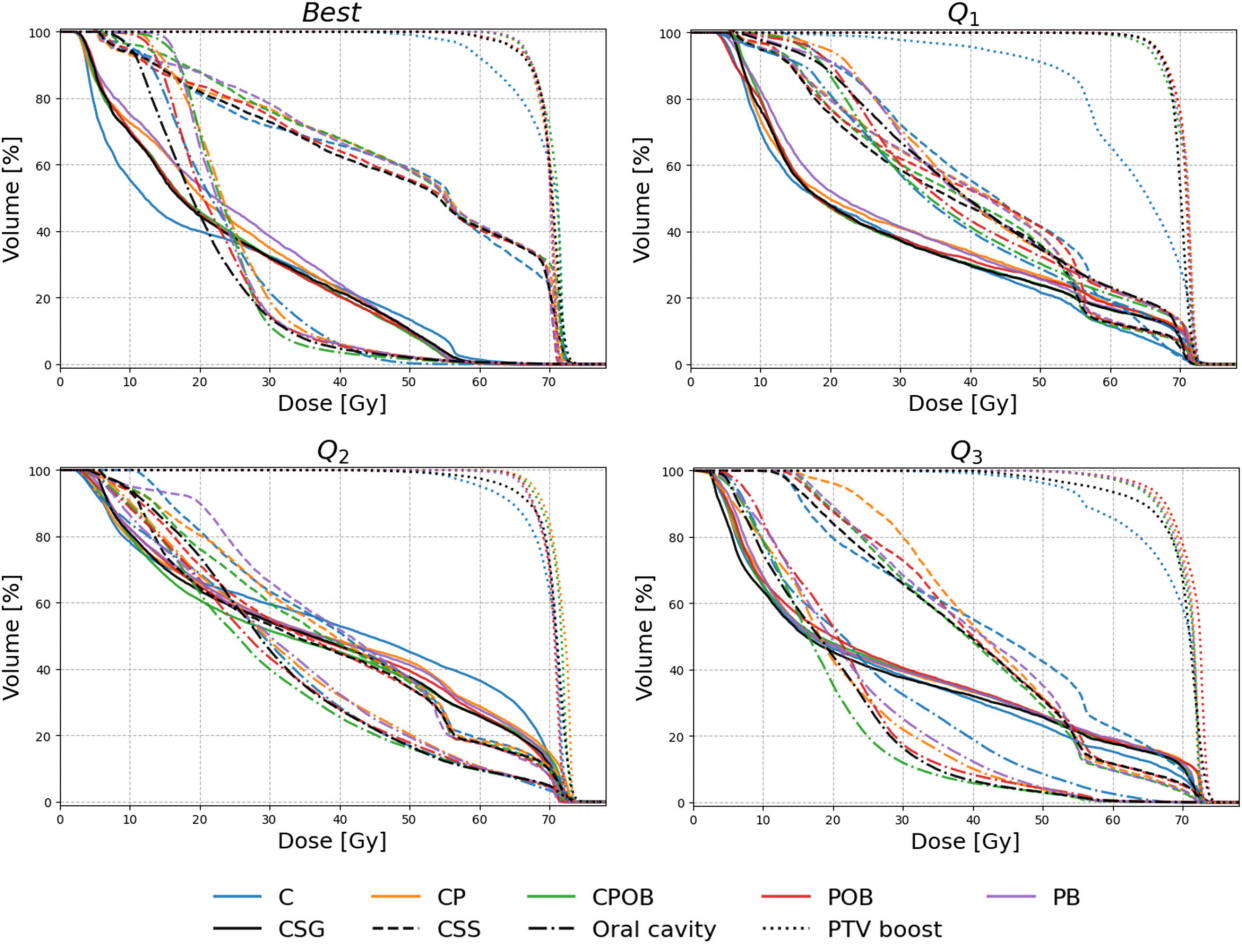
Dose volume histogram (DVH) of four selected patients for the models C, CP, CPOB, POB, and PB. The patients were selected to show a range of performances of the CPOB mean absolute error (best/Q_1_/Q_2_/Q_3_) in the volume of interest (VOI: slices containing PTV-E with a 7.5-mm margin in the body contour). The clinical DVHs are in black color, and the predicted DVHs of the five models are in blue, orange, green, red, and purple, respectively, for composite salivary glands (CSG, solid), composite swallowing structures (CSS, dashed), oral cavity (dash-dotted), and PTV-B (dotted). DVHs for individual OARs and PTV-E for Q_1_ and Q_2_ can be found in **Figures 2** and **3**, **Supplementary Material**.

**Figure 3** shows the dose volume histograms (DVH) for all five models for four patients, selected from the following: the best case, q1 (lower quartile), q2 (median), and q3 (upper quartile) of the average MAE for CPOB in the VOI.

### Mean dose to OARs

**Figure 4:**
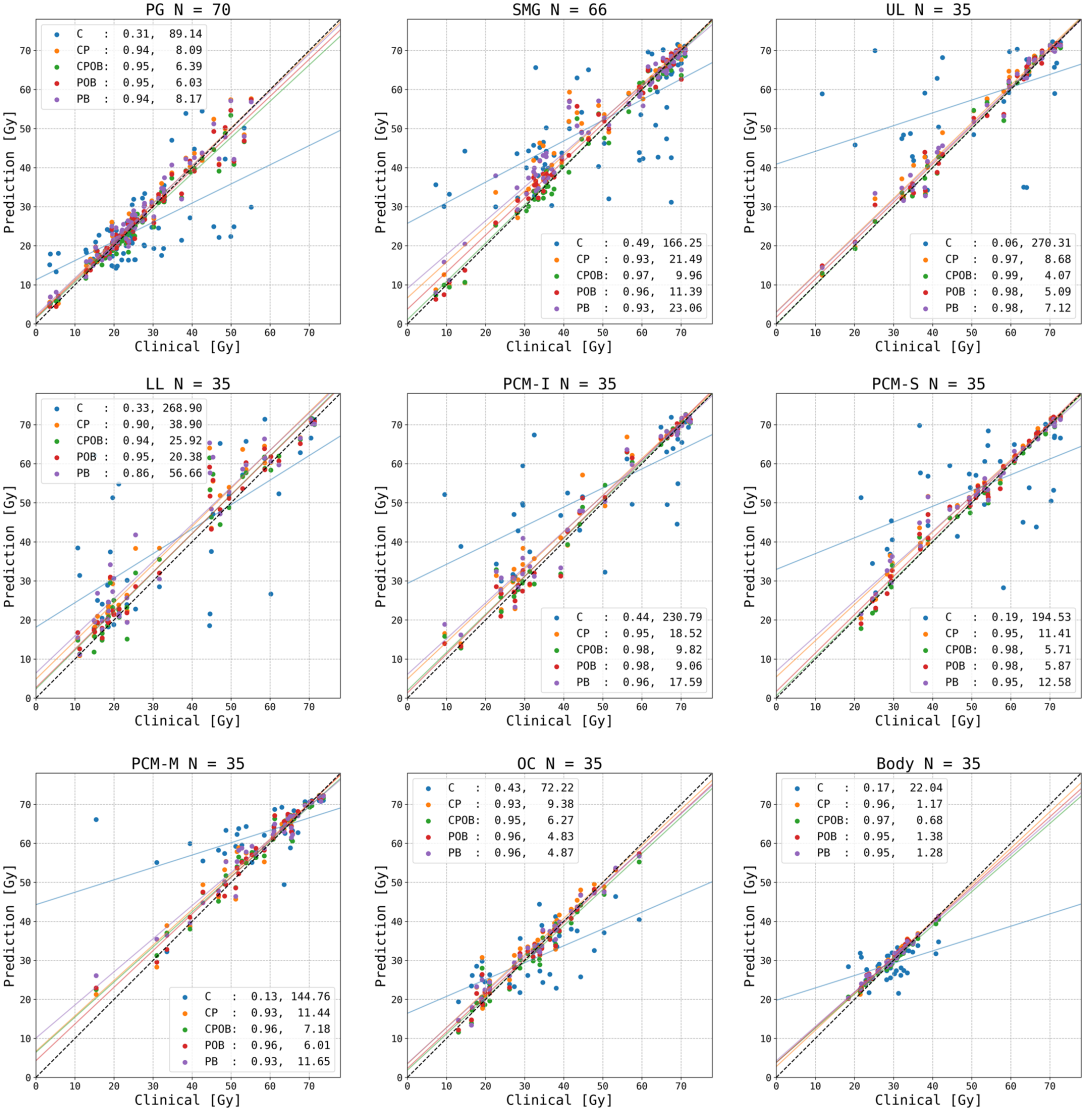
Predicted versus clinical mean dose to organs at risk (OARs). From left upper to right lower: parotid gland (PG) left and right combined, submandibular gland (SMG) left and right combined, upper larynx (UL), lower larynx (LL), oral cavity (OC), inferior pharyngeal constrictor muscle (PCM-I), medial pharyngeal constrictor muscle (PCM-M), superior pharyngeal constrictor muscle (PCM-S), and the entire body contour. Each data point represents the dose for one OAR for each of the 35 patients in the test set. N indicates the number of clinical contoured OARs. The vertical distance to the diagonal line shows the error between the predicted and clinical mean doses. For each model, an R^2^ correlation (left) and a residual measured in mean squared error (right) are in the legend. The colored lines are the regression lines for mean dose of each model.

**Figure 4** shows the mean dose per structure compared with the clinical mean doses, where none of the OARs above were trained and tested individually. The R^2^ scores indicate that the predictions were highly correlated with the clinical dose distributions; the mean squared errors illustrate the spread of the predictions. Models CPOB and POB had the highest correlation and the lowest spread for all OARs. CPOB achieved for most patients a predicted mean dose within ±3 Gy from the clinical dose, 82.6%, 88.6%, and 82.9% for all OARs, PG, and SMG, respectively, whereas POB had for most patients a deviation within ±6 Gy: 95.1%, 95.7%, and 94.3% for all OARs, PG, and SMG, respectively.

### Mean dose error

**Figure 5:**
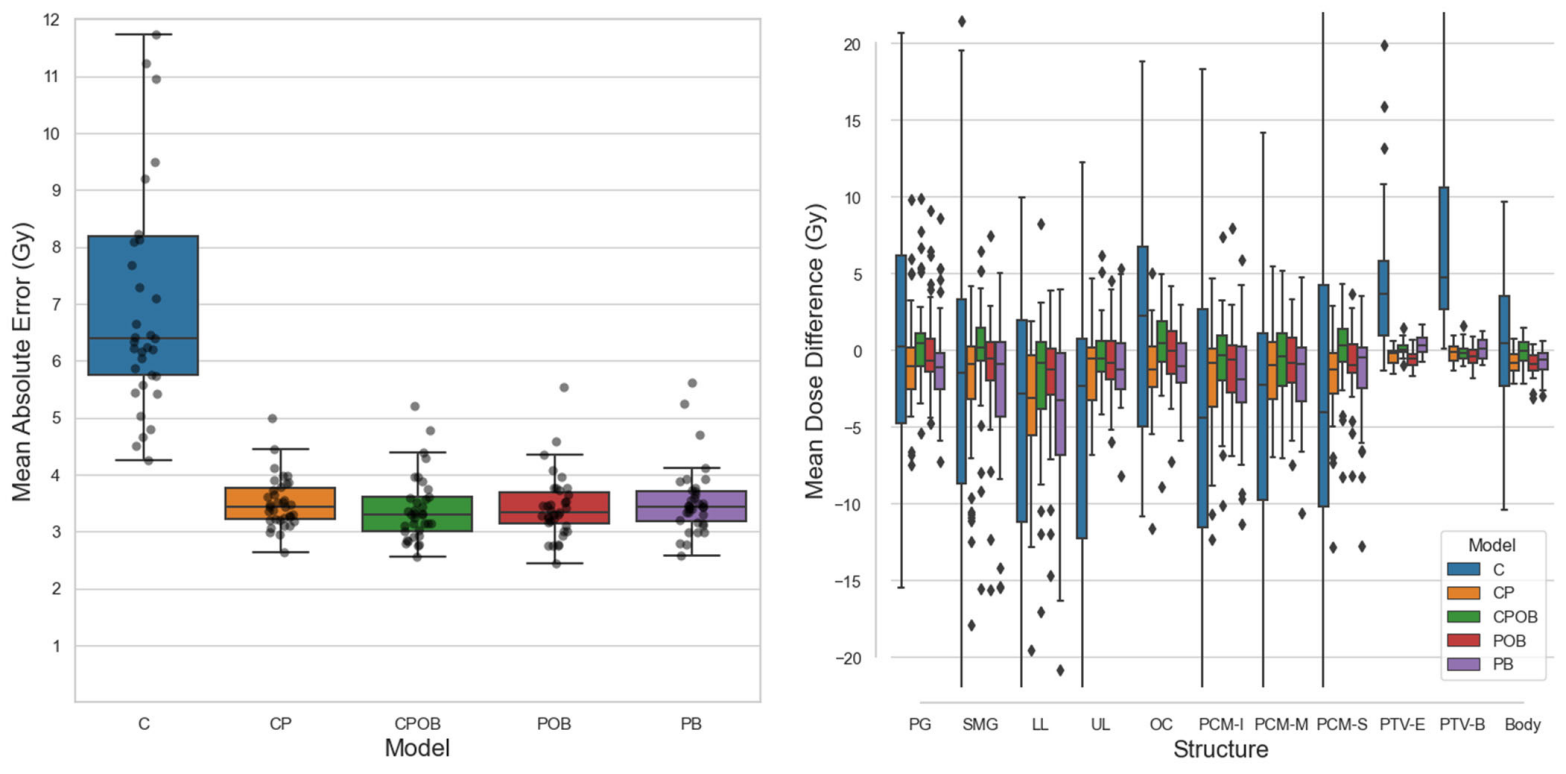
**(A)** (Left): Mean absolute dose error (MAE) over all voxels in the volume of interest for all five models on the test set. Mean values of each boxplot were 7.3, 3.5, 3.4, 3.4, and 3.5 Gy. Each dot represents a patient in the test set. The vertical axis shows the voxel-wise MAE between the clinical and predicted doses. In the boxplots, the lower and upper whiskers indicate the 1.5× the interquartile range<Q1 and >Q3, respectively, the data points outside the whiskers are considered outliers. **(B)** (Right): Mean dose differences (clinical – predicted) of individual structures. CPOB had the lowest mean dose differences for the OARs: 2.8, 2.8, 4.6, 2.7, 4.1, 3.1, 3.1, and 2.7 Gy. G, parotid gland (left and right combined); SMG, submandibular gland (left and right combined); LL, lower larynx; UL, upper larynx; OC, oral cavity: PCM-I, inferior pharyngeal constrictor muscle; medial pharyngeal constrictor muscle; PCM-S, superior pharyngeal constrictor muscle.

**Figure 5A** shows the mean dose difference between the clinical and predicted doses for different structures. The greatest differences were produced by model C where it was clear that if the model could not predict the correct tumor extent, it was impossible to predict the correct dose distribution resulting in PTV mean doses that were too low and incorrect OAR mean doses. The other four models showed a mean dose difference close to 0 and less spread. Models CP and PB, the models without OARs, led to doses in the OARs higher than in clinical plans, whereas models CPOB and POB, the models with OARs, more accurately predicted OAR doses. **Figure 5B** shows the MAE of the dose in all the voxels in the VOI, which excludes voxels outside the body and voxels in slices away from PTV-E, whereas the mean absolute dose errors over the entire dose distribution volume, i.e., 128 × 128 × 64 voxels, of the five models were 2.45, 1.35, 1.32, 1.31, and 1.35 Gy, respectively. Excluding model C, the remaining four models had comparable results. Model CPOB trained with the most comprehensive input data had both the lowest mean and median and performed significantly better than model PB (*p* = 0.007, **Supplementary Material Table 2**). Although model C has in general a very high MAE, there are a few patients with much lower MAE where this model manages to predict reasonable dose distributions. For the models trained with PTVs, the predicted PTV-E and PTV-B had minimal mean dose differences and the coverage was comparable with the clinical plans (**Figure 3**).

**Figure 6:**
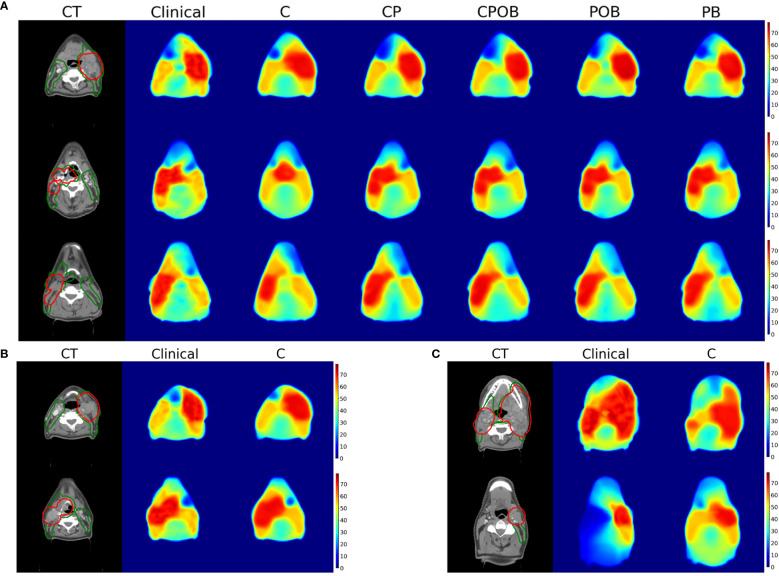
**(A)** (Up): Dose prediction comparison for a single slice for 3 patients with mean absolute dose errors in the volume of interest for model CPOB around the mean (3.62, 3.32, and 3.29 Gy). From left to right, CT with PTV-elective (green) and PTV-boost (red) contours, dose distributions of clinical, models C, CP, CPOB, POB, and PB. The CT scans above are displayed in the original resolution, which is better than the input to the models. **(B)** (Down-Left): Dose distributions predicted by model C of two patients with a small MAE in VOI (4.7 and 4.8 Gy). **(C)** (Down-right): Dose distributions predicted by model C of two patients with a large MAE in VOI (11.2 and 12.4 Gy). CT, computed tomography; OAR, organ at risk; PTV, planning target volume; MAE, mean absolute error; VOI, volume of interest.

**Figure 6A** shows examples of the predicted in comparison with the clinical dose distributions for three patients. There are notable differences between CT only and other results, whereas the differences among the other four models are small. For most patients, model C (CT only) was able to find the location of the tumor but was often inaccurate in predicting the extension of the PTVs. **Figures 6B**, **C** demonstrate examples of predicted dose distributions by model C with low and high MAEs in the VOI, respectively.

## Discussion

Deep learning for radiotherapy dose prediction has been reported for different tumor sites, but to the best of our knowledge, the influence of multiple different levels of input information on the ability to predict dose has not been (adequately) investigated. We demonstrated that all four models that included PTV contours predicted dose distributions that had a high level of agreement with clinical treatment plans. Although model CPOB, trained with the most comprehensive input data, produced the best dose predictions, even model CP, trained with patient CTs and primary tumor and lymph node PTVs and without explicit prior knowledge of the relevant OARs, achieved results that were not significantly different than those from the best model. The results and significance tests showed that for head and neck cancer, use of CT scans in training and testing adds little to dose prediction when OARs or PTVs are also used in model training. Somewhat surprisingly, using only the PTVs and the body contour (model PB) provided sufficient information for dose prediction, and presumably the shapes of the dose distributions were learnt and CT scans had limited added value for model training.

The mean dose to OARs and the shape of the DVHs demonstrated that the majority of predictions of all models except model C were consistently in line with the clinical doses and the PTVs. From the composite OAR structures, the models CPOB and POB had explicitly learned the OAR locations and sizes and more accurately predicted doses both in the individual OARs and in the VOI than the other three models. Although on average the predicted OAR doses were comparable with the clinical doses, there were individual patients with up to 17 Gy higher predicted dose than clinical, e.g., for the SMG that clinically received 41.4 Gy (**Figure 4**). All models predicted too high doses. This SMG was partly inside the PTV-B; the dose gradient was not steep enough, unlike the patients in **Figure 6A**. The overprediction could be caused by the downsampling resulting in larger voxel sizes, or by the fact that training samples from 2013 to 2015 had a less aggressive SMG sparing than in later years. Model POB and PB predicted comparable results as other models trained with the CT. As expected, the OARs provided significant information for OAR sparing (*p* = .015, **Supplementary Material Table 2**); however, for the model PB, with no knowledge of CT nor OARs, it had comparable results for both the DVH and mean dose to OARs.

While most other research using deep learning for dose prediction has used CT scans together with PTVs and OARs (14, 20), similar to our model CPOB, we observed from models CP and CPOB that when the CT was present in training and testing, the OAR contours did not result a statistically significant influence (*p* = .175, **Supplementary Material Table 2**) in the VOI. However, when the CT was absent, OAR structures made a statistically significant difference (*p* = .015, **Supplementary Material Table 2**) of the dose prediction for model POB over PB. There are limited published data demonstrating that NNs are capable of dose prediction without the need of the CT scans (17, 30). The results suggest that sufficient information had already been distilled from the CT in the OAR contours to predict dose distributions close to the clinically accepted ones. When CT scans are excluded from the training, the data distribution of PTV and OAR contours becomes binary resulting in a lower complexity and stronger gradients to update the parameters in the NNs. When the OARs are not part of the input, the model can learn their location and size from the CT scan and the PTV but seems to do this only to a limited extent (CP results were worse than CPOB). The results of the PB model indicated that the model has implicitly learned the typical location of OAR and the locations where the dose needs a steeper gradient, only from the PTV and body contours. The reason that this model performed slightly worse than model POB is probably that the exact location and size of the OAR cannot be estimated for individual patients.

When CT was the only input for our architecture, the majority of the dose predictions were not adequate. Large deviations in PTV-B were observed in the DVH, and predicted dose distributions of all 35 test patients assumed a bilateral PTV-E, which resulted in a high mean dose error when the PTV-E was unilateral, probably because most training cases had a bilateral PTV-E. Although the model seemed to recognize the location of the primary tumor for 25 out of 35 test patients based on visual inspection, it had difficulties in detecting from only the CT scan the extent of the tumor and which lymph node levels needed to be included for elective irradiation. In a minority of cases (8 out of 35 test patients, with lowest MAEs, **Figure 5A**), the model was able to better locate the tumor position and extent and the predicted-clinical DVH were in closer agreement. While others have investigated dose prediction using CT scans only and achieved good results (21, 24), this was for rectum and prostate cancer. Both have rather less complex PTVs than HNC and fewer OARs. For HNC, the extent of lymph node irradiation depends on the size and location of primary tumor and the presence of positive lymph nodes are more difficult to determine from only a CT scan. The performance of a model without any contours could possibly be improved with the addition of magnetic resonance imaging (MRI) data, which in general better shows the extent of the tumor. The enhanced tissue-tumor contrast of MRI could also provide extra information, and different types of MRI scans can highlight different types of tissues that could help the models to detect gross tumor volume (GTV). Including other modalities such as positron emission tomography (PET) can further improve GTV segmentation (31). In practice, incorporating MRI may pose challenges, including that (1) MRIs may not be available for all patients; (2) many centers do not acquire MRIs in the same position as the planning CTs, which makes registration more difficult; (3) clinical MRIs may not image the same volume as the CTs and may not image the entire PTV; and (4) there are many possible MRI sequences, delivering all differences in images, and different sequences may have been used for different patients.

We used composite OAR structures to better generalize the DVH analysis for individual patients. Plans often need to make a trade-off between OARs to spare, e.g., parotid or submandibular glands, sparing at the cost of the oral cavity and pharyngeal constructor muscles. Without this knowledge for individual patients, the use of composite structures may represent the overall quality of the dose prediction better than if individual OARs had been used, and it requires fewer input channels for training. Over time, OAR sparing has evolved in complexity and now includes many more OARs as the focus changes from sparing only the parotid glands to also minimizing the risk of damage to the submandibular glands, to reduce xerostomia and minimize the dose to the swallowing apparatus in order to reduce dysphagia. Even without using individual OARs for training and testing, the predicted mean doses evaluated on individual OARs were close to those of the clinical plans (**Figure 5**). For the best model, only 17.4% of all OARs deviated by more than 3 Gy from the clinical plans. This is better than the dose prediction using RapidPlan, a knowledge-based planning system, where 22.5% of head and neck OAR deviated by more than 3 Gy (27). The variations in our deep learning dose prediction were also substantially lower than the variation in planning between radiation therapy centers (3).

Models C, CP, and PB had to learn the position of OARs implicitly from the clinical dose distributions. Using two individual NNs for OAR segmentation and dose prediction could achieve better performance, because the weights of the NNs can be independent for the corresponding tasks. Since our study is meant to investigate the influence of input data for dose prediction, we have not yet experimented if our methods would benefit segmentation tasks. The models were trained with elastic loss function, a combination of the L1 and the L2 loss, which penalizes highly predicted doses on OARs in L2 loss’s quadratic term to encourage OAR sparing (for details, see **Supplementary Material**).

This study has its limitations. First, dose distribution has been predicted, but we have not shown how to convert this to a deliverable treatment plan. Second, the dataset was cropped and had larger voxel sizes than the original. This could be particularly a limitation for smaller OARs. However, we have not tested the influence of voxel size. Third, we used the central 96 slices in this study, with the length of 24 cm, such that on average 5% of OARs were excluded from the dataset, although the greatest dose differences were not observed at the caudal end. Furthermore, the clinical plans in the training and validation sets were drawn from a time span of 7 years, and the ability of OAR sparing may have changed overtime (32). As it was not known how many cases were needed for training, we opted for a sufficiently large training set, which necessitated the long period. Finally, none of the clinical plans used for training and testing were curated to ensure an optimal OAR sparing. Having a curated, consistent training and test set could possibly improve the models (and facilitate a smaller dataset). However, we have assumed that by having a sufficiently large training set, we could mitigate this.

Our model CP, trained only with CT scans and PTVs, was not statistically different from model CPOB, which was trained with OAR contours. This represents the minimum data statistically required to adequately predict the clinical dose distribution. This paper takes one step forward in (1) understanding how AI dose prediction works (i.e., what input data is important) and (2) achieving fully autonomous AI generated head and neck treatment plans, which could help to overcome limitations in time, manpower, experience, and financing. All the clinician need to do is to generate GTV/CTV boost (33), and CTV elective and OARs can be automatically generated (34, 35).

## Conclusions

In this study, we used deep generative adversarial networks to predict dose distributions for head and neck radiotherapy treatment planning and achieved results that were highly similar to the clinical plans. We demonstrated the influence of the CT scan and PTV and OAR contours and showed that CT scans give limited additional benefit when OARs were used; PTVs provide sufficient information for OAR sparing; and models trained together with OARs have the lowest mean absolute dose differences. Our model CP, trained only with CT scans and PTVs, was not statistically different from model CPOB, which was trained with OAR contours.

## Data availability statement

The original contributions presented in the study are included in the article/**Supplementary Material**, further inquiries can be directed to the corresponding author.

## Ethics statement

This study was reviewed by the Medical Ethics Review Committee of VU University Medical Center. Due to the retrospective nature of this study which does not include tests or different treatments on human individuals, and the use of anonymized data, it was concluded by the Medical Ethics Review Committee that the Medical Research involving Human Subjects Act (WMO) does not apply to this study and that official approval of this study by the committee is not required. The study was conducted in accordance with the local legislation and institutional requirements.

## Author contributions

XG and VS contributed equally to this work and share first authorship. All authors contributed to the article and approved the submitted version.
