# Supplementary material for: Dose distribution prediction for head-and-neck cancer radiotherapy using a generative adversarial network: influence of input data
Source: Front Oncol. 2023 Sep 26;13:1251132. doi: 10.3389/fonc.2023.1251132 (PMC10565853; doi:10.3389/fonc.2023.1251132)
Supplement: Supplementary file 1 [file DataSheet_1.pdf]

| Model | Input Data | CT | PTVs | OARs | Body |
|---|---|---|---|---|---|
| C | CT | X | | | |
| CP | CT + PTVs | X | X | | |
| CPOB | CT + PTVs + OARs + Body | X | X | X | X |
| POB | PTVs + OARs + Body | | X | X | X |
| PB | PTVs + Body | | X | | X |

**Suppl.Mat. Table 1:** Overview of the experimental outline. *Abbreviations*: CT: computed tomography; PTV: planning target volume; OAR: organ at risk.

| *p*-value | C | CP | CPOB | POB | PB |
|---|---|---|---|---|---|
| C | | **<.001** | **<.001** | **<.001** | **<.001** |
| CP | **<.001** | | 0.175 | 0.444 | >.999 |
| CPOB | **<.001** | 0.175 | | >.999 | **0.007** |
| POB | **<.001** | 0.444 | >.999 | | **0.015** |
| PB | **<.001** | >.999 | **0.007** | **0.015** | |

**Suppl.Mat. Table 2:** Significance test of mean absolute dose error (MAE) in the volume of interest (VOI) between models. Wilcoxon significance test was used for each model tested against the other four models, α=0.05, with a Bonferroni correction per set of tests to adjust the p-values. Bold ones are significant.

| Model | PG | SMG | LL | UL | OC | PCM-I | PCM-M | PCM-S | Body |
|---|---|---|---|---|---|---|---|---|---|
| C | 0.31<br>89.14 | 0.49<br>162.22 | 0.33<br>268.9 | 0.06<br>270.31 | 0.43<br>72.22 | 0.44<br>230.79 | 0.13<br>144.76 | 0.19<br>194.53 | 0.17<br>22.04 |
| CP | 0.94<br>8.09 | 0.93<br>20.86 | 0.90<br>38.9 | 0.97<br>8.68 | 0.93<br>9.38 | 0.95<br>18.52 | 0.93<br>11.44 | 0.95<br>11.41 | 0.96<br>1.17 |
| CPOB | 0.95<br>6.39 | **0.97**<br>**9.79** | 0.94<br>25.92 | **0.99**<br>**4.07** | 0.95<br>6.27 | 0.98<br>9.82 | 0.96<br>7.18 | **0.98**<br>**5.71** | **0.97**<br>**0.68** |
| POB | **0.95**<br>**6.03** | 0.97<br>10.91 | **0.95**<br>**20.38** | 0.98<br>5.09 | **0.96**<br>**4.83** | **0.98**<br>**9.06** | **0.96**<br>**6.01** | 0.98<br>5.87 | 0.95<br>1.38 |
| PB | 0.94<br>8.17 | 0.93<br>22.55 | 0.86<br>56.66 | 0.98<br>7.12 | 0.96<br>4.87 | 0.96<br>17.59 | 0.93<br>11.65 | 0.95<br>12.58 | 0.95<br>1.28 |

**Suppl.Mat. Table 3**: Correlation between the predicted mean dose to structures and the clinical doses. For each score, the upper is R-squared correlation, the lower is the residual. The last column contains the mean R-squared scores and residual of 9 structures. Bold ones are the best out of the five models. *Abbreviations*: PG: parotid gland; LL: lower larynx; UL: upper larynx; OC: oral cavity; SMG: submandibular gland; PCM-I: inferior pharyngeal constrictor muscle; PCM-M: medial pharyngeal constrictor muscle; PCM-S: superior pharyngeal constrictor muscle.

**Network architecture**

The generator has six levels of downsizing convolution and six levels of upsizing transposed convolution blocks. In downsizing, the number of channels starts from 32 and doubles in every level to compensate the smaller image sizes after max pooling, and the number of layers reaches 2048 in the middle bottle neck layer; then the number of layers halves in every upsizing convolution block. Each convolution block contains two convolution layers to increase the number of learnable parameters. In each upsizing block an additional learnable transposed convolution layers was used. Leaky ReLU activation function was chosen during downsizing used to for better feature extraction; and during upsizing, ReLU activation was chosen to reduce noise in reconstructed dose distributions. The last convolutional layer outputs a single channel unbounded dose distribution (-inf, inf), a sigmoid function bounds the values to (0, 1), and the values are scaled to (0, 80) in post-processing. The

discriminator has 4 levels of downsizing convolution and followed by a sigmoid activation function for binary classification.

**Elastic loss**

Motivated by the elastic net regularization(1), the smooth L1 loss(2) and the Huber loss(3), we used a weighted combination of the L1 (mean absolute error) and L2 (mean squared error) loss functions named as elastic loss: $L_{elastic} = \lambda_1|y - \hat{y}| + \lambda_2\,(y - \hat{y})^2$, where y and ŷ are the clinical and predicted dose distributions, respectively, and the weighting hyperparameters were chosen as $\lambda_1 = 1$ and $\lambda_2 = 0.5$, which was the same as the naïve choice in elastic net, we have not explored other values in this study, since these values have surpassed L1 and L2 loss functions alone. The elastic loss penalizes the outliers in quadratic term, as a voxel-wise loss function, it penalizes the voxels with high dose in the organs at risk (OARs) to encourage OAR sparing.

Unlike the L1 loss, elastic loss is differentiable everywhere, the loss value and the gradient of the loss are both 0 when the error, i.e., $y - \hat{y}$, is 0. Furthermore, when the prediction is close to the ground truth, or when the range of ground truth is within [-1, 1], L2 loss produces smaller loss values such that the gradient for learning is also smaller, resulting in a slower learning.

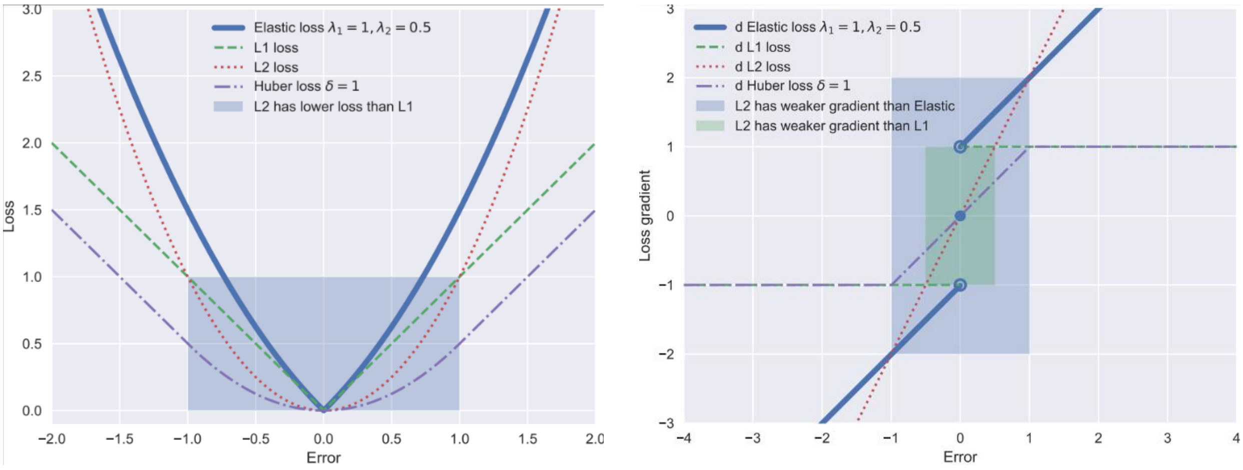


**Suppl.Mat. Fig 1**: **Left:** Comparison of elastic, L1, L2 and Huber loss functions. **Right:** Comparison of the gradient of elastic, L1, L2 and Huber loss functions.

Figure 1.Left compares the element-wise loss among the loss functions, where for the Elastic loss $\lambda1 = 1$, $\lambda2 = 0.5$ is chosen, and for the Huber loss $\delta = 1$ is chosen, i.e. the Smooth L1 loss. The Elastic loss penalizes the differences in quadratic form, similar to the L2 loss. It also has a steeper slope towards the minimum, whereas the L2 loss has a mellow bowl shape under the threshold of 1. Consequently, the L2 loss has a lower loss value in the shaded area.

Figure 1.Right compares the gradients among the loss functions. In the green shaded area, the L2 loss has a weaker gradient than the L1 loss when the loss is in [−0.5, 0.5], while in the blue shaded area, the L2 loss has a weaker gradient than the Elastic loss, when the loss is in [−1, 1]. The Elastic loss has substantially stronger gradient than the L2 loss in these areas, and also the L1 loss is a better loss function when the absolute error is less than 0.5. When the target is normalized in [0, 1], using the L2 loss will most likely to produce the worst result; whereas when the target is normalized in [−1, 1], the increased error can benefit the L2 loss at the beginning of the training.

## Dose Volume Histograms

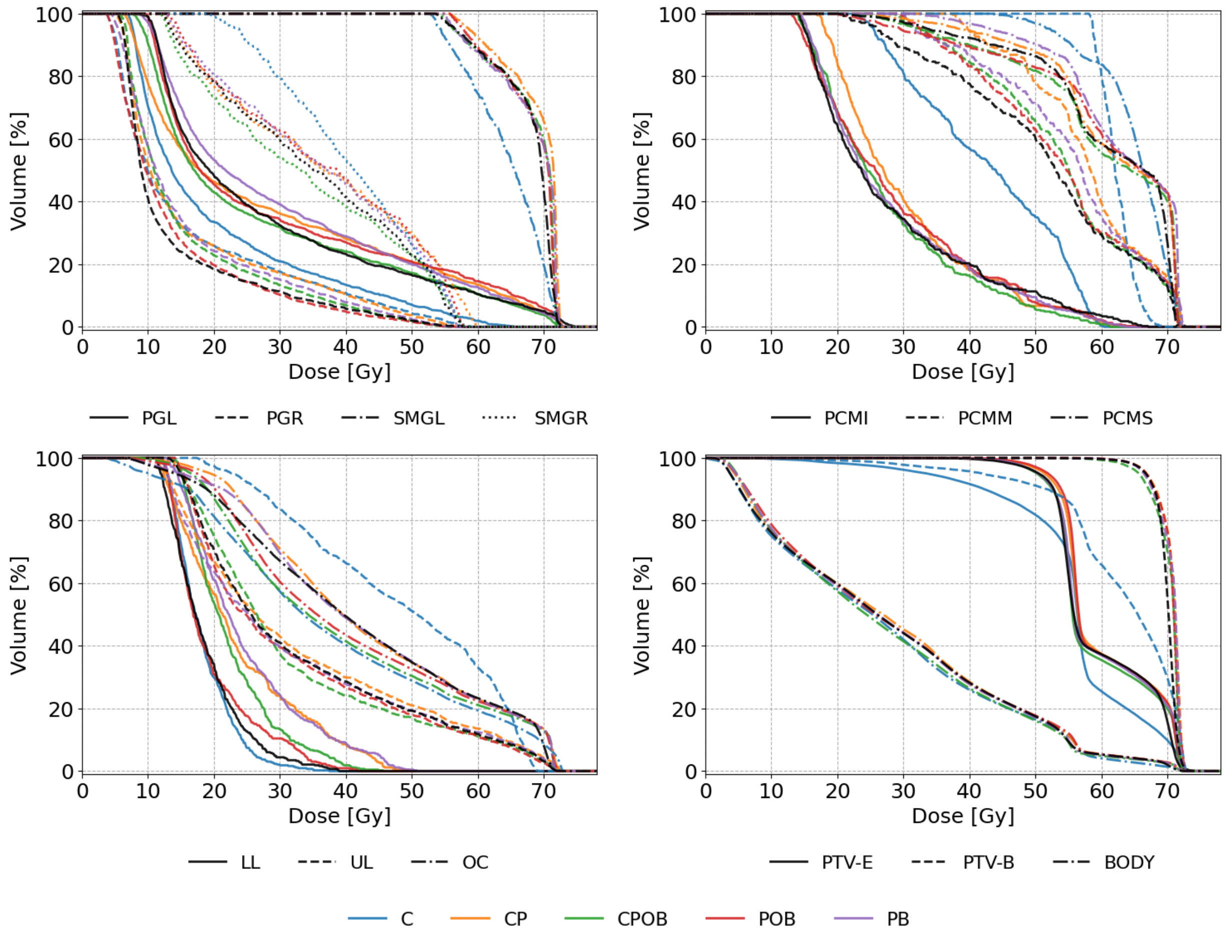


**Suppl.Mat Fig 2**: Dose volume histogram of individual structures of the patient at Q1 (lower quartile), based on the mean absolute error in the volume of interest. *Abbreviations*: PGL: parotid gland left; PGR: parotid gland right; SMGL: submandibular gland left; SMGR: submandibular gland right; PCMI: inferior pharyngeal constrictor muscle; PCMM: medial pharyngeal constrictor muscle; PCMS: superior pharyngeal constrictor muscle; LL: lower larynx; UL: upper larynx; OC: oral cavity; PTV-E: elective planning target volume elective; PTV-B: boost planning target volume.

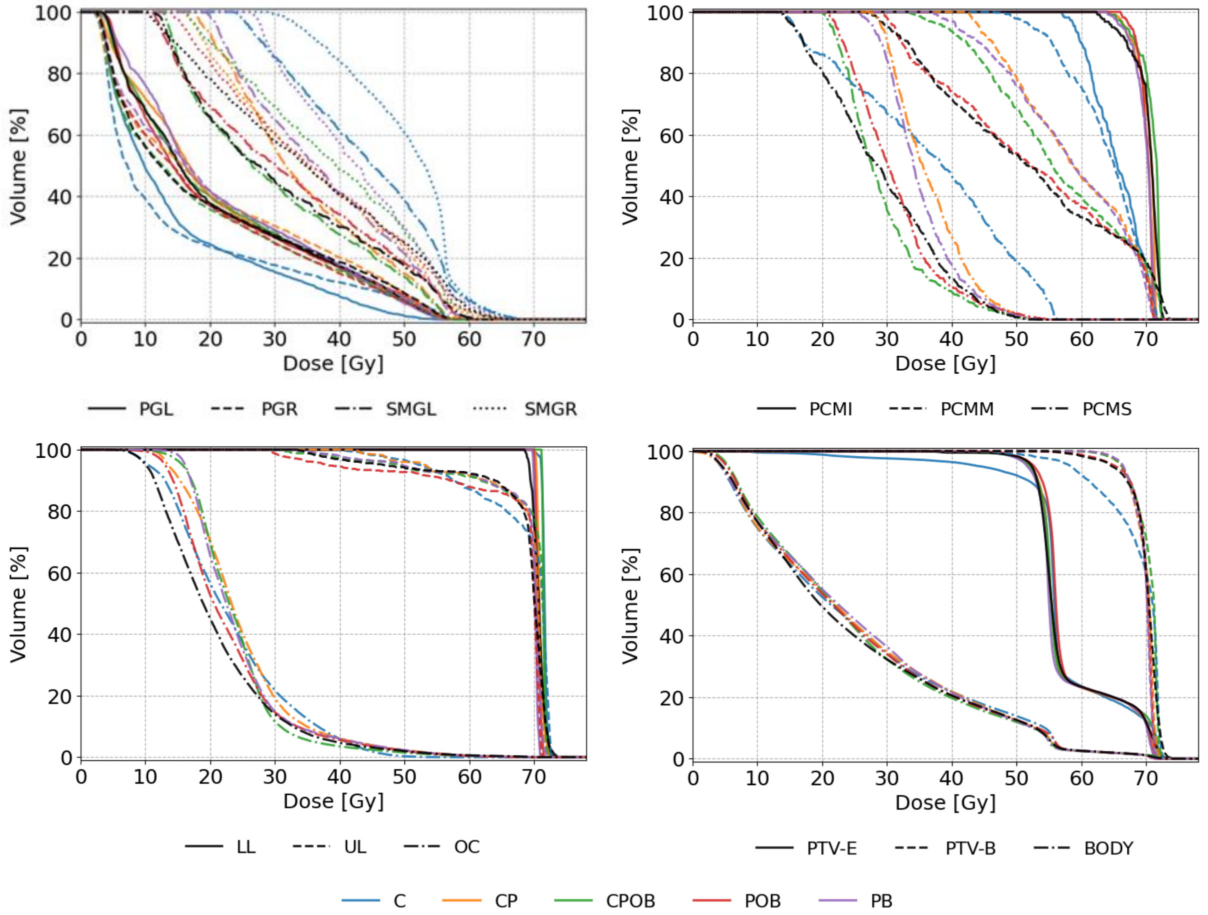


**Suppl.Mat Fig 3**: Dose volume histogram of individual structures of the patient at Q2 (median), based on the mean absolute error in the volume of interest. *Abbreviations*: PGL: parotid gland left; PGR: parotid gland right; SMGL: submandibular gland left; SMGR: submandibular gland right; PCMI: inferior pharyngeal constrictor muscle; PCMM: medial pharyngeal constrictor muscle; PCMS: superior pharyngeal constrictor muscle; LL: lower larynx; UL: upper larynx; OC: oral cavity; PTV-E: elective planning target volume elective; PTV-B: boost planning target volume.

Suppl.Mat Figures 2 and 3 show the dose volume histograms of 2 out of four selected patients in Figure 2. The OARs shown were not used training and testing, the composite structures were used.

## References


1. Zou H, Hastie T. Regularization and variable selection via the elastic net (journal of the royal statistical society. Series b: Statistical methodology (2005) 67 (301-320)). *Journal of the Royal Statistical Society Series B: Statistical Methodology* 2005;67:768-768.
2. Girshick R. Fast r-cnn. *Proceedings of the IEEE International Conference on Computer Vision* 2015;2015 Inter:1440-1448.
3. Huber PJ. Robust estimation of a location parameter. *The Annals of Mathematical Statistics* 1964;35:73-101.
